# Consequences of Shift Work and Night Work: A Literature Review

**DOI:** 10.3390/healthcare11101410

**Published:** 2023-05-12

**Authors:** Isabel Silva, Daniela Costa

**Affiliations:** 1 Interdisciplinary Centre of Social Sciences (CICS.NOVA.UMinho), University of Minho, 4710-057 Braga, Portugal; 2 School of Psychology (EPsi-UMinho), University of Minho, 4710-057 Braga, Portugal

**Keywords:** shift work, night work, health, family life, organizational context

## Abstract

Nonstandard work schedules such as shift work and night work tend to trigger problems for workers in different areas. To illustrate the diversity of areas affected and the relative interest of the scientific community, we conducted a literature review of the effects of shift work and night work on workers. In particular, we intended to identify the main variables addressed in the field of health, the family sphere, and the organizational context. The literature review was carried out using the Web of Science with the following terms: “shift work”, “rotating shifts”, and “night work”. Inclusion criteria incorporated empirical studies and articles written in Portuguese or English published in 2019. We selected 129 of the 619 articles identified. Regarding the impacts of shift work and night work, there existed a high discrepancy of focus between the three defined areas: health, family life, and organizational context. Specifically, health-related variables were the most studied (83.4%), followed by organizational variables (9.2%), and, lastly, family variables (7.4%). Based on these results, it is essential to extend the study of the two underrepresented impacts to other crucial areas, not only for the worker but also for organizations.

## 1. Introduction

Following the industrial revolution, societies began to organize themselves according to the time that individuals dedicated to work in a dichotomy of “time inside work” versus “time outside work” [1]. Until the middle of the 20th century, this working time was almost exclusively translated into conventional working hours (i.e., working on weekdays from morning to afternoon/evening, with rest on the weekend) [2]. However, with the evolution of societies, technology, and the economy, organizations were faced with the need to increase working time, in some cases extending the working time to 24 h a day, 7 days a week, leading to the growth of nonstandard work schedules [3,4]. Such arrangements include, among others, shift work, night work, evening work, and weekend work.

Shift work tends to be defined as an “organization of daily working hours in which different teams work in succession to cover more or all of the 24 h” [5] (p. 89). According to the Sixth European Working Conditions Survey of the EU-28 in 2015, 19% of workers performed night work, and 21% worked in shifts [6]. In the USA, according to the National Health Interview Survey (NHIS 2015), about 26% of workers worked in shifts, whether in the evening, at night, or on rotating shifts [7]. However, shift work and night work have been identified in the literature as the most harmful schedules for workers [3,8], and their negative impacts can translate into three primary domains: health, family and social life, and the organizational context.

In terms of health, problems tend to arise due to the circadian disturbance to which the workers are subjected. When shift work involves working at night, it requires an inversion of the workers’ sleep–wake cycle, which can lead to disturbances in their circadian systems [9,10]. This has been associated with several health problems. Specifically, the literature has identified several health problems associated with shift work and night work, including sleep problems [11], cardiovascular problems [12,13], psychological problems [14], oncological problems [15,16], and problems with the female reproductive system [17,18]. For example, in the meta-analysis by Yuan et al. [16], a positive relationship was found between long-term night work and the risk of breast cancer, digestive cancer, and skin cancer. The authors also found that the risk of breast cancer increased by 3.3% for every five years of night work. In cardiovascular terms, Ahmadi et al. [12] found differences in the DNA methylation of circadian genes between shift workers and day workers. This circadian disruption may be related to increased cardiometabolic risk and, consequently, cardiovascular disease. Additionally, in an integrative literature review conducted in 2020, it was revealed that shift workers were more likely to have sleep problems, fatigue, depression, and burnout [11].

In the family and social domain, shift work requires working during periods that are highly valued from a family and social point of view, such as evenings, nights, and/or weekends. Temporal desynchronization with the family and/or society can trigger conflict between the two spheres of an individual’s life (work and family) [19,20]. This conflict can lead to problems in family relationships and/or consequences for other members of the household. In fact, the literature has found negative relationships between shift work and the work–family interface [21,22], children’s well-being [20], and marital satisfaction [23], among others. For example, in a study by Zhao et al. [22] involving 452 fathers and 756 mothers, shift work was associated with higher work–family conflict. These irregular work schedules were also positively related to psychological distress either directly or indirectly, in this case through work–family conflict. Furthermore, in the literature review by Li et al. [20], parents’ nonstandard work schedules were associated with negative child developmental outcomes (e.g., mental health issues, obesity, and behavioral problems). Parental depressive symptoms, low-quality parenting, and a less favorable home environment were some of the factors that could provoke this negative association.

In the organizational context, the literature has found an association between shift work, especially night work, and variables such as safety, productivity, or worker absenteeism. These associations began to gain greater attention when it was realized that accidents with major impacts, such as the 1986 Chernobyl nuclear accident in Ukraine, occur mostly during night shifts [5,24]. In terms of safety, some studies [25,26] have pointed to a greater risk of occupational accidents occurring when there is shift work, at night, or during long work shifts (e.g., 12 h shifts). Moreover, the performance of employees may be substandard when they work in shifts [24,27]. In the literature review by Dall’Ora et al. [27], working in rotating shifts was associated with degraded job performance, while working at night on a fixed (non-rotating) basis seemed to allow workers resynchronization. However, the demands that these work schedules cause in terms of physical and emotional exhaustion can contribute to increased sick leave [28,29], although not necessarily in a direct way. For example, in the study by Jacobsen and Fjeldbraaten [29], carried out in a Norwegian hospital, it was observed that there was no direct effect of shift work on absenteeism, but this effect was mediated by work–family conflict and perceived health.

The various domains associated with shift and night work can interact with each other [29,30,31,32], so this issue gains greater relevance when such interdependence occurs. For example, in a study by Fonseca et al. [31], carried out in Portugal, burnout was positively related to sleep problems and problems with family life. Likewise, in the literature review of Bastos and Afonso [30], shift work was associated with several health problems, such as sleep problems, mental health problems, and cognitive impairment, which can consequently increase the number of medical errors.

Considering that the issue of shift work can give rise to a multiplicity of negative impacts, not only on the workers themselves, but also in the family and organizational context, the main purpose of this review was to identify the variables studied by the scientific community to understand the various impacts of shift work and night work schedules and their relative interest in terms of frequency. Based on the established purpose, the following research questions were defined:

Q1: What kind of variables (and respective domains) are studied to understand the impacts of shift work and night work?

Q2: What is the relative interest in the variables identified by the scientific community in terms of the frequency of these variables in the analyzed studies?

Although literature reviews on the impact of shift and night work are relatively common, particularly in a specific domain (e.g., sleep, cardiovascular, or oncological problems), simultaneous analysis of the various impacts and respective frequencies is scarce.

## 2. Materials and Methods

The search for articles was carried out using the Web of Science database, with the year 2019 as the time interval. This interval was chosen because it was the year immediately preceding the emergence of COVID-19, so its use avoids any possible biases resulting from the pandemic in the study of this topic. The search terms were established with the goal of reaching all studies carried out on the intended work schedules (i.e., rotating shift work schedules, including nights and fixed night shifts), thus the following terms were researched: “Shift work”, “Shiftwork”, “Rotating shifts”, “Night shifts”.

The inclusion criteria used in the research were:

1. Journal articles;
2. Studies in Portuguese or English;
3. Empirical studies;
4. Studies where rotating shift work or a fixed night shift was the main objective.

In turn, the exclusion criteria were:

1. Studies not related to shift and night work schedules (first phase of elimination by reading the title and abstract);
2. Studies that did not evaluate the effects of shift and night work schedules (different work schedules, for example, rotating without nights);
3. Studies in which shift work or night work appeared as a secondary objective (risk factor or mediating variable);
4. Theoretical articles (literature reviews, non-empirical articles, or validation of scales);
5. Meta-analyses;
6. Laboratory studies (in this case, the participants were not shift workers and the work schedule was simulated).

### 2.1. Analysis and Selection of Studies

**Figure 1:**
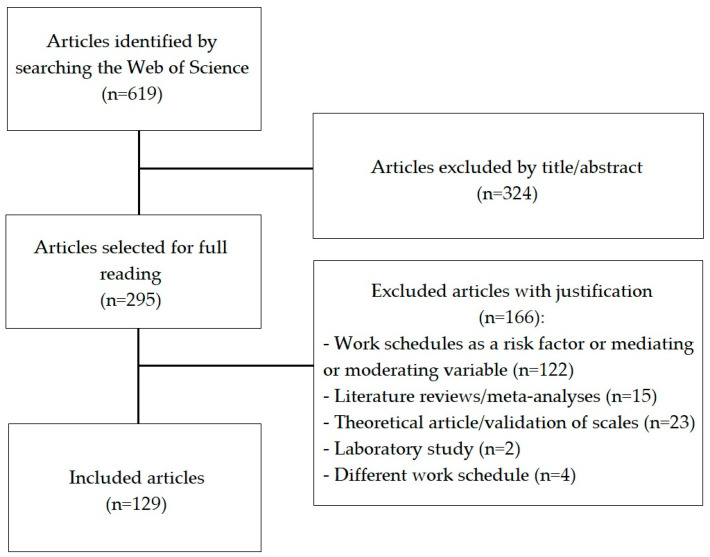
Flowchart of the article selection process.

The search resulted in the identification of 619 articles. In the first reading of the titles and abstracts, 324 articles were eliminated, as their subject matter was different from the intended purpose. With the full reading of the remaining 295 articles, 166 articles were eliminated because they did not meet the inclusion criteria for the following reasons: (i) in 122 articles, shift work and night shift were not the main objectives of the study (i.e., work schedules were used as a mediating or moderating variable or appeared as a risk factor); (ii) 40 articles were not empirical studies (i.e., they were literature reviews or meta-analyses, theoretical articles, or laboratory simulations); and (iii) in four articles, the nonstandard work schedules were different from shift work or fixed night work. In total, 129 articles were analyzed (Figure 1).

### 2.2. Analysis and Categorization of Variables

The 129 articles included in the analysis were analyzed using Content Analysis [33]. Previously, and based on the literature review [3], three main areas were established to categorize the impacts of work schedules: health, family and social life, and organizational context. Then, after the reading and analysis of each article, different subcategories were identified for each category. For example, in the health category, one of the problems mentioned was oncological problems, with these problems constituting the “Oncological” subcategory. In order to have a more detailed analysis of the type of problem being studied, each subcategory was also subject, when necessary, to division according to variables. In the example given, the subcategory “Oncological” was divided into “Breast cancer”, “Prostate cancer”, “Ovarian cancer”, and “Carcinogens agents”.

## 3. Results

Of the 129 studies that researched relationships with work schedules and other variables, most were conducted in Europe (35.7%), North America (28.7%), and Asia (27.9%). The most representative country was the USA (15.5%), followed by South Korea (10.1%). The researchers were mostly (86.8%) affiliated with Health Sciences, such as Medicine, Nursing, Epidemiology, or Nutrition. As for the sectors of activity of shift workers, health (47.3%) and industry (14.7%) were the most representative sectors. About 18% of the studies only addressed fixed night work schedules, while the majority of the studies studied the impacts of shift work. More than 66% of the 129 studies studied samples consisting entirely or mostly of women. Samples ranged from 10 to 631,418 participants.

Table 1 presents the results obtained in the analysis carried out. Taking into account the nature of the variables, and in order to systematize the information, they were divided into three main categories: health, organizational, and family. Next, each main category was divided into subcategories and, when justified, in each subcategory the variables were made explicit. At the level of the main category “Health”, 136 references emerged (83.4% of the categories). For the organizational context, 15 references emerged (9.2% of the categories), and at the family level, 12 references emerged (7.4% of the categories). It should be noted that the total number of references assigned to the main categories (n = 163) is greater than the number of selected articles (n = 129) because in some articles there was a reference to more than one category. For example, as we can see in Table 1, the study by Burch et al. [34] was associated with the category “Health”, but within this main category, it was associated with two subcategories: sleep and fatigue and cardiovascular. So, in this case, there are two references to the same article.

In terms of health, sleep and fatigue were the most studied variables in the literature (n = 27), with studies related to sleep [34,35,36,37,38,39,40,41,42,43,44,45,46,47,48,49,50,51,52,53,54] predominating on the topic of fatigue [34,38,44,55,56,57]. The second most referenced subcategory was lifestyles (n = 19), where eating habits stood out [58,59,60,61,62,63,64,65,66], followed by physical exercise [66,67,68], alcoholism [67,69], smoking [67,68], and health behaviors [71,72]. The cardiovascular system references (n = 17) were divided into cardio-metabolic risk factors [65,73,74,75,76,77,78,79,80,81,82], hypertension [81,83,84,85], and heart rate [34,86]. The subcategory related to mental health included 16 references, highlighting the study of aspects such as anxiety and depression [37,50,55,87,88] and burnout [41,84,89]. In the metabolic system subcategory (n = 13), there was an emphasis on the problems of the system itself [94,95,96,97,98,99,100], obesity [60,104], and diabetes [85,101,102,103]. The association between breast cancer [105,106,107,108,109,110] or prostate cancer [111,112] and work schedules was studied several times, representing a total of 10 references to oncological problems. Both the immune system [50,115,116,117,118,119], as well as well-being and quality of life [69,71,120,121,122,123], had six references each. The female reproductive system is mainly divided into studies related to miscarriage [127], menopause [128], and pregnancy [124,125,126]. Three studies addressed aging [129,130,131], while two studies focused on the digestive system [132,133]. Finally, studies relating to variables that appeared only once were included in a subcategory entitled “Others”. As an example, the following variables studied in connection with shift work and night work are mentioned: eyesight [134], all-cause mortality [136], body image [138], respiratory system [139], multiple sclerosis [141], and hearing [144].

The second main category, related to the organizational context (n = 15), was mainly divided into three subcategories: performance [34,42,54,145,146,147,148], absenteeism [149,150,151,152], and safety [153,154,155]. Finally, only one study related the workload [135] to shift work.

Finally, the third main category was mainly divided into impacts at the level of three systems: children, spouses/partners, and family, totaling 12 references. At the level of children, most studies [156,157,158,159] linked parents’ work schedules with children’s health or well-being. At the family level, the work–family conflict was the most studied variable [88,162]. Finally, regarding spouses, satisfaction [162] and marital relationship [161] were studied.

## 4. Discussion

The purpose of this study was to identify the variables used in the literature in the study of shift and night work schedules and their relative interest in terms of frequency. In this sense, two research questions were defined: (i) What kind of variables (and respective domains) are studied in understanding the impacts of shift work and night work? and (ii) What is the relative interest in the variables identified in terms of its frequency by the scientific community in understanding the impacts of shift work and night work? The literature review showed a substantial difference in the number of references between the three defined domains, with a clear predominance in the study of variables related to health as compared to variables related to the family context and the organizational context. The finding of this discrepancy is not new [163], and has persisted over the last few years [164]. Indeed, in the bibliometric study by Sweileh [164], all articles on shift work that appeared in the Scopus database from 1 January 2012 to 31 December 2021 were analyzed. The results indicated that health problems such as sleep, fatigue, circadian rhythm, and circadian disruption were the main topics studied in the field of shift work over the last decade. In addition, it was observed that the most studied professionals were from the health sector, in particular, nursing.

This literature review indicates a wide variety of impacts of shift and night work on the worker’s physical and psychological health, covering problems related to sleep, the cardiovascular system, the metabolic system, the female reproductive system, oncological problems, or anxious and depressive symptoms, among others. Overall, these results are consistent with previous literature reviews [13,14,16]. Given the evidence of the impact of shift work and night work on workers’ health, increased efforts should be made to minimize such impacts. Measures such as reducing the number of consecutive night shifts, having a sufficient interval between shifts, and reducing the duration of night shifts can help reduce the circadian disruption of workers, consequently reducing impacts on health [165].

At the level of the family system and the organizational context, globally, the results found in this review lean in the direction of the difficulties mentioned in a period prior to the target year of analysis, namely at the marital [23] and parental levels [20] and, in organizational terms, at the levels of safety [25] and performance [27]. Given the interdependence between the various domains involved, the management of shift work and night work must rely on a holistic approach. For example, in a study carried out in the North American context, it was observed that workers working nonstandard hours, in order to maximize time with their children, spent less time with their spouse, slept less, and dedicated less time to their leisure activities, such as watching television [32]. Additionally, the study by Ganesan et al. [42], night work was associated with less alertness and worse performance due to a lack of circadian adaptation.

Despite the relevance of complaints of a social nature related to shift work and night work, this review indicates that these have received less interest from the scientific community. This lack of interest is not recent. At the beginning of the 21st century, Smith et al. [163] (p. 179) had already noted, based on the literature available at the time, that “this dearth of research is surprising, given that the most frequent complaint of shift workers is that shiftwork interferes with their personal lives”. Some reasons have, however, been advanced in the literature for understanding this situation: (i) a smaller number of studies published by social science researchers [166]. In fact, in the present work, only 13% of the studies were conducted by researchers in the field of social sciences and humanities; (ii) the high degree of complexity of family and social life [166]; (iii) the fact that when studies are carried out at the level of the work–family relationship, the indirect effects on household members are rarely part of the research, though some authors [23,167] have been stressing the importance of including these perspectives in the study of this theme. In fact, in a recent study conducted in a Portuguese context, partners of police officers who worked shifts, when compared to partners of police officers who worked normal hours, reported a significantly greater interference of their spouse’s work schedule in the organization of family life, marital relationships, and parental relationships [161]. The various ongoing changes, such as demographic (e.g., aging of the active population), societal (e.g., greater participation of women in the labor market and the increase in the number of dual-earner couples), and economic and technological (e.g., globalization of the economy, teleworking), will certainly contribute to greater diversification and intensification of nonstandard work schedules. Therefore, these changes call for the need to deepen the study of the impacts of a family and social nature, not only from the perspective of the workers themselves, but also from the perspective of third parties (e.g., spouses and children). In this context, for example, the report Working Time and Work–life Balance around the World by the International Labour Organization [168] concluded that the deepening of the study of the work–family balance related to work schedules is important since such balance can bring benefits to workers and employers.

The first limitation of the work is the fact that the analysis examined a time interval of only one year. Despite this limitation, it should be noted that the results found are, roughly speaking, consistent with a study that used a longer time interval [164]. On the other hand, the clear predominance of the health sector in the identified articles (43.7% of the articles were in this sector, followed by industry with 14.7%) presupposes the need to extend the study of the impacts of work schedules to other sectors of activity, particularly those that may be less represented. Another discrepancy observed in the analyzed studies is related to the composition of the samples in terms of gender, with more than two-thirds of the studies applying to women. Additionally, it is equally important to address this imbalance in the study of the impacts of shift and night work.

## 5. Conclusions

This review reinforces the impact that shift and night work can have on multiple dimensions of worker health. It is, therefore, important to continue efforts to understand the consequences in this area. In addition, the review highlights the need to deepen the study of the impacts of shift work and night work on other variables of the organizational context and the family/social context. Concerning such contexts, the almost complete absence of perspectives from third parties (e.g., children, spouses, and managers) is also highlighted. Given that the impacts of shift work and night work are not limited to the workers themselves, but also involve family, social, and organizational domains, it is recommended that such a perspective be integrated in order to establish a multifaceted understanding of the impacts of this way of organizing working time.

## Figures and Tables

**Table 1:** Categories, subcategories, and variables used in studying the effects of shift work and night work.

Main Category	Subcategories	Variables	Articles	
Health (n = 136)	Sleep and fatigue (n = 27)	Sleep (n = 21)	Burch et al. [34] Cerqueira Teixeira et al. [35] Chang and Li [36] Dai et al. [37] Di Muzio et al. [38] B. Ferguson et al. [39] Flaa et al. [40] Fowler and Ellis [41] Ganesan et al. [42] Haile et al. [43] Härmä et al. [44]	Hattammaru et al. [45] Hulsegge, Loef et al. [46] Jeong et al. [47] Mulhall et al. [48] Pallensen et al. [49] H. Park et al. [50] Resuehr et al. [51] Schuster et al. [52] Uekata et al. [53] Wilson et al. [54]
		Fatigue (n = 6)	Burch et al. [34] Di Muzio et al. [38] Härmä et al. [44]	Bazazan, Rasoulzadeh et al. [55] Chang et al. [56] Yu et al. [57]
	Lifestyle (n = 19)	Eating habits (n = 9)	Müge Arslan et al. [58] Fradkin et al. [59] Fröhlich et al. [60] Heath et al. [61] Nogueira et al. [62]	Peplonska et al. [63] Shaw et al. [64] Terada et al. [65] van de Langenberg et al. [66]
		Physical exercise (n = 3)	van de Langenberg et al. [66] Buchvold et al. [67]	Kolbe-Alexander et al. [68]
		Alcoholism (n = 2)	Buchvold et al. [67]	Pham and Park [69]
		Smoking (n = 2)	Buchvold et al. [67]	Y.M. Cho et al. [70]
		Health behaviors (n = 2)	Kędzierska et al. [71]	Navarro et al. [72]
		Caffeine (n = 1)	Buchvold et al. [67]	
	Cardiovascular (n = 17)	Cardiometabolic risk factors (n = 11)	Terada et al. [65] Hermansson et al. [73] Holst et al. [74] Hulsegge, Picavet et al. [75] Joo et al. [76] Kang et al. [77]	Kwak et al. [78] Ritonja et al. [79] Skogstad et al. [80] Strzemecka and Skrodziuk [81] Tucker et al. [82]
		Hypertension (n = 4)	Strzemecka and Skrodziuk [81] J.M. Ferguson et al. [83]	Nascimento et al. [84] J. Park et al. [85]
		Heart rate (n = 2)	Burch et al. [34]	Muzeyyen Arslan et al. [86]
	Mental health (n = 16)	Depression (n = 5)	Dai et al. [37] H. Park et al. [50] Bazazan, Rasoulzadeh et al. [55]	Hammer, Hageman et al. [87] Moreira et al. [88]
		Anxiety (n = 3)	Dai et al. [37] Bazazan, Rasoulzadeh et al. [55]	Moreira et al. [88]
		Burnout (n = 3)	Fowler and Ellis [41] Nascimento et al. [84]	Peterson et al. [89]
		Suicidal ideation (n = 2)	Kim et al. [90]	Park [91]
		Psychological health (n = 1)	Terada et al. [65]	
		Antidepressant prescriptions (n = 1)	Hall et al. [92]	
		Resilience (n = 1)	Tahghighi et al. [93]	
	Metabolic system (n = 13)	System problems (n = 7)	Bracci, Copertaro et al. [94] Gowda et al. [95] Khosravipour et al. [96] Kiranmala et al. [97] Ledda et al. [98]	Loef, van Baarle, van der Beek, Beekhof et al. [99] Nikpour, Tirgar, Hajiahmadi, Hosseini et al. [100]
		Diabetes (n = 4)	J. Park et al. [85] Hanprathet et al. [101]	Silva-Costa et al. [102] Zoto et al. [103]
		Obesity (n = 2)	Fröhlich et al. [60]	Rabanipour et al. [104]
	Oncological (n = 10)	Breast cancer (n = 6)	Bracci, Ciarapica et al. [105] Bustamante-Montes et al. [106] Carugno et al. [107]	Jones et al. [108] Pahwa et al. [109] Pham et al. [110]
		Prostate cancer (n = 2)	Barul et al. [111]	S. Cho et al. [112]
		Ovarian cancer (n = 1)	Leung et al. [113]	
		Carcinogens agents (n = 1)	El-Zaemey and Carey [114]	
	Immune system (n = 6)		H. Park et al. [50] Hanprathet et al. [115] Loef, Nanlohy et al. [116]	Nikpour, Tirgar, Hajiahmadi, Ebadi et al. [117] Reinhardt et al. [118] Teixeira et al. [119]
	Well-being and quality of life (n = 6)	Well-being (n = 4)	Kędzierska et al. [71] Haluza et al. [120]	Imes and Chasens [121] Levin et al. [122]
		Quality of life (n = 2)	Pham and Park [69]	Turchi et al. [123]
	Female reproductive system (n = 5)	Pregnancy (n = 3)	Clarkson-Townsend et al. [124] Specht et al. [125]	Willis et al. [126]
		Miscarriage (n = 1)	Begtrup et al. [127]	
		Menopause (n = 1)	Stock et al. [128]	
	Aging (n = 3)		Y.I. Choi et al. [129] Nabe-Nielsen et al. [130]	White et al. [131]
	Digestive system (n = 2)		H. Choi et al. [132]	F. Wang et al. [133]
	Others (n = 12)	Hematological system (n = 1)	Cerqueira Teixeira et al. [35]	
		Eyesight (n = 1)	Abrishami et al. [134]	
		Musculoskeletal diseases (n = 1)	Bazazan, Dianat et al. [135]	
		All-cause mortality (n = 1)	Hannerz et al. [136]	
		Pain (n = 1)	Katsifaraki et al. [137]	
		Body image (n = 1)	Leite et al. [138]	
		Respiratory system (n = 1)	Loef, van Baarle, van der Beek, Sanders et al. [139]	
		Skin (n = 1)	Lu et al. [140]	
		Multiple sclerosis (n = 1)	Papantoniou et al. [141]	
		Endocrine system (n = 1)	Razavi et al. [142]	
		Urinary system (n = 1)	Sigalos et al. [143]	
		Hearing (n = 1)	D. Wang et al. [144]	
Organizational context (n = 15)	Performance (n = 7)		Burch et al. [34] Ganesan et al. [42] Wilson et al. [54] Behrens et al. [145]	Patterson et al. [146] Rosa et al. [147] Tummers et al. [148]
	Absenteeism (n = 4)		Dall’Ora et al. [149] Hammer, Garde et al. [150]	Ropponen et al. [151] Vedaa, Pallesen et al. [152]
	Safety (n = 3)		Donnelly et al. [153] Nielsen et al. [154]	Vedaa, Harris et al. [155]
	Workload (n = 1)		Bazazan, Dianat et al. [135]	
Family life (n = 12)	Children (n = 7)	Children’s health or well-being (n = 4)	Kaiser et al. [156] Strohmaier, Devore, Huang et al. [157]	Strohmaier, Devore, Vetter et al. [158] Wei et al. [159]
		Parenthood (n = 2)	Moreira et al. [88]	Matheson et al. [160]
		Parental relationship (n = 1)	Costa and Silva [161]	
	Family (n = 3)	Work–Family Conflict (n = 2)	Moreira et al. [88]	Vaghar and Masrour [162]
		Family environment (n = 1)	Costa and Silva [161]	
	Partners (n = 2)	Marital relationship (n = 1)	Costa and Silva [161]	
		Marital satisfaction (n = 1)	Vaghar and Masrour [162]	

Note: The number of references assigned to the main categories (n = 163) is greater than the number of selected articles (n = 129) because in some articles there is a reference to more than one subcategory.

## Data Availability

Not applicable.
